# The Toll-like Receptor 4 Polymorphism Asp299Gly Is Associated with an Increased Risk of Ovarian Cancer

**DOI:** 10.3390/cells11193137

**Published:** 2022-10-05

**Authors:** Katarzyna D. Kania, Daria Haręża, Jacek R. Wilczyński, Miłosz Wilczyński, Dariusz Jarych, Andrzej Malinowski, Edyta Paradowska

**Affiliations:** 1 Laboratory of Virology, Institute of Medical Biology, Polish Academy of Sciences, 93-232 Lodz, Poland; 2 Bio-Med-Chem Doctoral School and Institutes of the Polish Academy of Sciences, University of Lodz, 90-237 Lodz, Poland; 3 Department of Surgical and Oncological Gynecology, Medical University of Lodz, 90-419 Lodz, Poland; 4 Department of Gynecology and Obstetrics, Tomaszow Health Center, 97-200 Tomaszow Mazowiecki, Poland; 5 Department of Surgical, Endoscopic and Oncological Gynecology, Polish Mother’s Health Center Research Institute, 93-338 Lodz, Poland

**Keywords:** toll-like receptor, single-nucleotide polymorphism, ovarian cancer, human papillomavirus

## Abstract

Ovarian cancer (OC) is one of the most common cancers threatening women’s lives around the world. Epithelial ovarian tumors represent the most common ovarian neoplasms. Most OC patients are diagnosed at the advanced stage, and there is an urgent need to identify novel biomarkers of the disease. Single-nucleotide polymorphisms (SNPs) in *TLR* genes may serve as crucial markers of cancer susceptibility. We investigated the frequency of TLR polymorphisms in a group of 200 women, including 70 with OC. Four SNPs, two each in *TLR4* (rs4986790 and rs4986791) and *TLR9* (rs187084 and rs5743836), were analyzed using polymerase chain reaction–restriction fragment length polymorphism (PCR-RFLP). The digested fragments were separated and identified by multicapillary electrophoresis. The load quantification of human papillomavirus (HPV) types 16/18 was determined using a digital droplet PCR method. We found an increased frequency of heterozygous genotype and minor allele of the *TLR4* rs4986790 SNP in women with OC compared with healthy controls, and this result remained highly significant after Bonferroni’s correction for multiple testing (*p* < 0.0001). No evidence of linkage disequilibrium was found with any of the examined *TLR* SNPs. The findings suggest that the *TLR4* Asp299Gly polymorphism could be a genetic risk factor for the development of OC.

## 1. Introduction

Ovarian cancer (OC) is a leading cause of cancer-related deaths in women [1]. There were almost 314,000 new cases of OC in 2020 worldwide, and it is estimated that this number will increase to 429,000 by 2040 [2]. About 75% of women with OC are diagnosed at the advanced stage of the disease because patients are often asymptomatic, while the 5-year relative survival rate is only 29% [3,4]. Most OC cases are high-grade serous ovarian carcinomas (HGSOCs), which are the most common and aggressive subtype of OC [5]. It can originate from precursor epithelial lesions in the fimbriated end of the fallopian tube (FT), although the genetic alterations and pathophysiological processes that drive the progression of cancer are unclear [6,7,8,9,10]. The mutations in the *BRCA1/2* and *TP53* genes are important risk factors for OC, as well as FT and primary peritoneal cancers [9,11]. In carriers of these mutations, occult malignancy of serous histology accompanied by intraepithelial carcinoma or dysplasia is frequently found in the fimbrial end of the FT [9,12]. Moreover, the presence of human papillomavirus (HPV) DNA in cancerous ovarian and FT tissues was found, although there is still no evidence that demonstrates an association between HPV infection and ovarian carcinogenesis [13,14,15]. However, there is still an urgent need to identify new biomarkers of this disease and evaluate their prognostic value.

Toll-like receptors (TLRs) are type I integral transmembrane proteins belonging to the family of pattern recognition receptors (PRRs), which play a crucial role in immune responses, especially pathogen recognition. These receptors are known to play a significant role in innate immunity and chronic inflammation. Increasing evidence suggests that TLRs are also important regulators of tumor biology that have either antitumor or protumor effects on carcinogenesis or tumor progression [16,17]. Among them, human TLR4 was the first discovered and reported to regulate inflammatory responses [18]. Endosomal TLR9 recognizes double-stranded DNA containing unmethylated cysteine–phosphate–guanine (CpG)-DNA motifs present in microbial nucleic acids [19]. Both receptors can initiate a signaling cascade involving NF-κB that culminates in the upregulation of proinflammatory pathways. TLR signaling promotes carcinogenesis via proinflammatory, antiapoptotic, proliferative, and profibrogenic signals within the tumor cells or tumor microenvironment (TME) [20]. TLR4 has also been demonstrated to promote the epithelial–mesenchymal transition (EMT) and cancer cell migration [21,22,23]. Both TME and inflammation are thought to be substantial for cancer initiation, development, and progression. Epithelial cells of the female reproductive tract may acquire carcinogenic changes through TLR stimulation by the pathogen-associated molecular patterns (PAMPs) [24]. The expression of TLRs is found in OC, where their activation seems to have tumor-promoting effects [24,25,26]. Ovarian cancer tissues demonstrated the upregulated expression of TLR4 at mRNA and protein levels compared to normal ovaries [25,27,28]. Furthermore, the upregulation of TLR4 was associated with a different histologic type and tumor progression [27,28,29,30]. Similarly, TLR9 expression increases with rising grades in OC [31]. In cervical cells, TLR9 expression levels are higher in women persistently infected with the same human papillomavirus (HPV) genotype with respect to women who cleared HPV infection [32]. Since genetic polymorphisms are known to affect cancer susceptibility, progression, and metastasis, it is vital to examine the associations between *TLR* SNPs and the development of OC. In the *TLR4* gene, the two most common polymorphisms, Asp299Gly (rs4986790) and Thr399Ile (rs4986791), are known to modify susceptibility to various human pathogens [33]. These polymorphisms seem to be related to the increased risk of different cancer types, including colorectal cancer [34,35], precancerous gastric lesions, and gastric cancer [36,37,38,39]. The role of *TLR9* gene polymorphisms has been studied in several diseases, including various cancers [40,41]. Most of them focused on three polymorphisms, including rs5743836, rs187084, and rs352140. Significant positive results were obtained for rs5743836 and Hodgkin’s lymphoma [42], as well as rs187084 and cervical carcinoma [43,44,45]. Moreover, rs352140 polymorphism was associated with an increased risk of cervical cancer in the presence of HPV16 infection [46]. However, there are no reports on the influence of *TLR4* and *TLR9* single-nucleotide polymorphisms (SNPs) on OC susceptibility.

It was hypothesized that the presence of *TLR* genotypes may lead to OC development, especially HGSOCs, and may be associated with HPV-related cases. Hence, the relevance of *TLR4* SNPs, Asp299Gly (rs4986790) and Thr399Ile (rs4986791), as well as two *TLR9* rs187084 and rs5743836 SNPs, were studied in 200 women, including 70 OC cases.

## 2. Materials and Methods

### 2.1. Clinical Samples

Seventy women with OC were enrolled in the study (median age: 62.5, range: 30–87 years). All of the patients underwent cytoreductive surgery at the Department of Surgical and Oncological Gynecology, the Medical University of Lodz, at the Department of Surgical, Endoscopic and Oncological Gynecology, Polish Mother’s Health Center Research Institute, Lodz, and at the Tomaszow Health Center, Poland. Among them, examined whole blood samples were obtained from 40 patients with high-grade serous ovarian carcinomas (HGSOCs), 19 ovarian cancers of other histologic types, and 11 women with metastatic OCs. Among the 19 women with other OC types, the following cancers were diagnosed: clear cell ovarian cancer (5 cases), borderline tumor of the ovary (BOT, 4 cases), endometrioid ovarian cancer (3 cases), adenocarcinoma mucinosum (3 cases), and 4 cases of other types. Pathologic diagnoses were established at the Department of Pathology, either the Medical University or Polish Mother’s Health Center Research Institute, Lodz, Poland. One hundred thirty healthy women were included in the control group. The peripheral blood samples (ca. 5 mL vol.) were collected from antecubital veins and stored at −80 °C until required for assays. The blood samples from OC patients were obtained during primary surgery. All individuals were enrolled from the central area of Poland and were Caucasian. The study was approved by the Ethics Committee of the Medical University of Lodz (RNN/346/17/KE and KE/1147/20) and was conducted according to the principles expressed in the Declaration of Helsinki and good clinical practice guidelines. Written informed consent was obtained from all participants before study entry.

### 2.2. Genotyping of TLRs Polymorphisms

**Figure 1:**
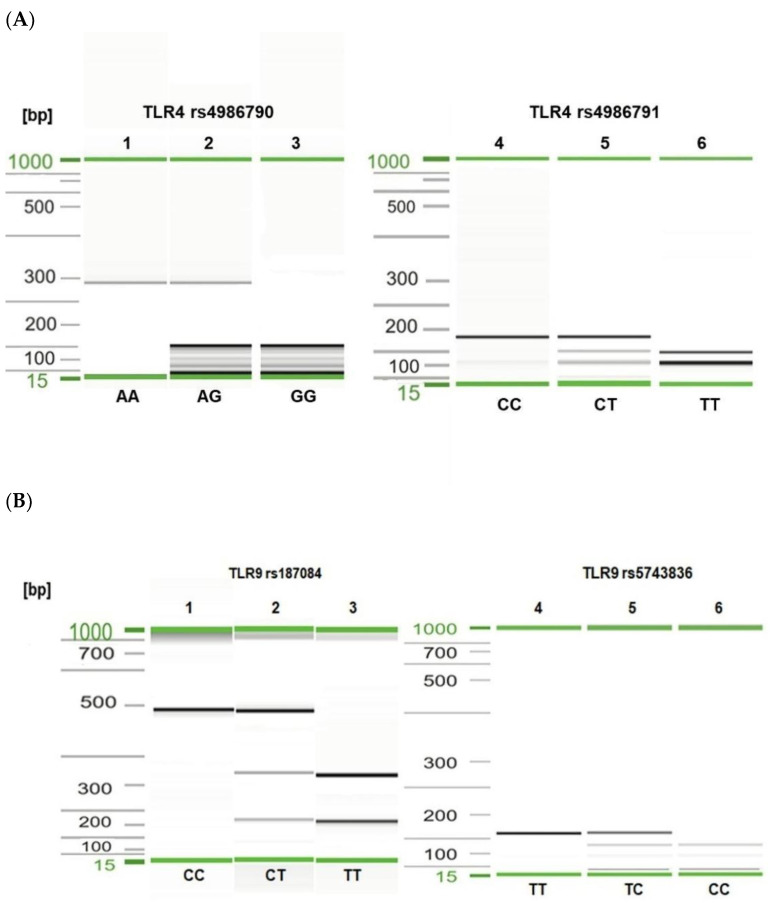
Visualization of selected PCR-RFLP products for *TLR4* rs4986790, rs4986791 (**A**) and *TLR9* rs187084, rs5743836 (**B**) genotyping. Gel image: (**A**), *TLR4* genotyping (1–3—rs4986790); 1, AA genotype; 2, heterozygous AG genotype; 3, GG genotype; (4–6—rs4986791); 4, CC genotype; 5, CT genotype; 6, TT genotype; (**B**), *TLR9* genotyping (1–3—rs187084); 1, CC genotype; 2, CT genotype; 3, TT genotype; (4–6—rs5743836); 4, TT genotype; 5, TC genotype; 6, CC genotype. Alignment markers (15 bp, 1 kbp).

The total genomic DNA was extracted from EDTA-anticoagulated peripheral blood using the DNeasy Blood & Tissue Kit (Qiagen, Hilden, Germany) according to the manufacturer’s protocol. The concentration and purity of DNA were estimated using a NanoDrop 2000c UV–vis Spectrophotometer (Thermo Scientific, Wilmington, DE, USA). A total of four SNPs in the *TLR4* (896A > G, rs4986790, Asp299Gly; 1196C > T, rs4986791, Thr399Ile) and *TLR9* (1486T > C, rs187084; 1237T > C, rs5743836) genes were determined and analyzed. The SNP selection was based on their possible functional effect and associations with infectious diseases. The molecular typing of *TLR* SNPs was performed by polymerase chain reaction–restriction fragment length polymorphism (PCR–RFLP) as described elsewhere [47,48,49,50]. PCR was carried out in a 50 μL mixture containing: 0.5 μg template DNA (5 μL), 5 μL 10 × DreamTaq™ Buffer (ThermoFisher, Vilnius, Lithuania), 5 μL 2.5 mM dNTP, 0.5 μL gene-specific primers (100 pmol/μL of each, Genomed, Warsaw, Poland), 0.25 μL DreamTaq™ Polymerase (5 U/μL); ThermoFisher), and nuclease-free water. The thermal cycling conditions for the *TLR4* gene fragment were 15 min at 95 °C and 35 cycles of 30 s at 94 °C, 30 s at 62 °C for rs4986790, or 30 s at 60 °C for rs4986791, and 30 s at 72 °C for both SNPs. The PCR parameters for *TLR9* rs5743836 were as follows: 4 min at 94 °C and 40 cycles each of 30 s at 95 °C, 20 s at 50 °C, and 30 s at 72 °C. The PCR parameters for *TLR9* rs187084 were 4 min at 95 °C and 35 cycles each of 30 s at 95 °C, 20 s at 60 °C, and 30 s at 72 °C. The reactions were performed in the Biometra TAdvanced thermal cycler (Analytik Jena Gmbh, Göttingen, Germany) and T100 Thermal Cycler (Bio-Rad, Hercules, CA, USA). The amplicons corresponding to the *TLR4* rs4986790 and rs4986791 and *TLR9* rs187084 and rs5743836 polymorphisms were digested with the restriction enzymes NcoI, HinfI, AflII, and MvaI, respectively (Thermo Scientific). The digested DNA fragments were separated and analyzed using the QIAxcel system (Qiagen). To determine the PCR-RFLP product sizes, the QX DNA Size Marker 50–800 bp and the BioCalculator software (Qiagen) were used (Figure 1). The samples of each *TLR* SNP were sequenced using the Sanger method to confirm the detected genotypes.

### 2.3. Quantification of TLR4 mRNA

The whole blood samples were drawn into PAXgene^®^ Blood RNA tubes (PreAnalytiX GmbH, Qiagen and BD, Hilden, Germany). The tubes were gently inverted, kept at room temperature overnight, and stored at −80 °C. The total RNA was extracted from frozen peripheral blood with a QIAamp RNA Blood Mini Kit (Qiagen) and the PAXgene^TM^ Blood RNAKit 50 (PreAnalytiX), according to the manufacturer’s instructions. cDNA for quantitative RT-PCR was synthesized using the High-Capacity cDNA Reverse Transcription Kit (Thermo Fisher Scientific, Inc., Vilnius, Lithuania). The relative transcription of the *TLR4* gene was determined by real-time PCR using Power SYBR™ Green PCR Master Mix (Thermo Fisher Scientific) and a 7900HT Fast Real-Time PCR System (Applied Biosystems, Foster City, CA, USA). The expression of the human *TLR4* gene (Gene ID: 7099; forward primer: AAGCCGAAAGGTGATTGTTG; reverse primer: CTGAGCAGGGTCTTCTCCAC) [51], as well as ACTB (Gene ID: 60; forward primer: AGAAAATCTGGCACCACACC; reverse primer: TAGCACAGCCTGGATAGCAA) and *GAPDH* (Gene ID: 2597; forward primer: AATGGGCAGCCGTTAGGAAA; reverse primer: GCCCAATACGACCAAATCAGAG), as reference genes were evaluated. For each sample, the relative expression level of the mRNA was calculated by the comparison with the control housekeeping ACTB/GAPDH genes using the 2^−∆∆Ct^ method. Each sample and non-template controls were run in duplicate.

### 2.4. Quantification of HPV16/18 DNA

The HPV16 and HPV18 viral load quantification in DNA isolates was determined using a digital droplet PCR (ddPCR) assay and the QX200 Droplet Digital PCR System (Bio-Rad Laboratories, Inc., Hercules, CA, USA). The specific primers and TaqMan probe sets for the HPV16 *E6* gene [52,53] or HPV18 *E7* gene [54] and the endogenous human RPP30 assay [55] were used. Subsequent amplification was performed in a T100 Thermal Cycler (Bio-Rad) with a ramp rate of 2 °C/s according to the following stages: 95 °C for 10 min followed by 45 cycles at 94 °C for 30 s and 60 °C for one minute, and 98 °C for 10 min. Droplets positive for FAM (HPV *E6/E7*) and HEX (*RPP30*) fluorescence were read in a QX200™ Droplet reader (Bio-Rad Laboratories, Inc.). The ddPCR data were analyzed using QuantaSoft™ Software Version 1.6.6. Manual thresholds were set for both HPV genotypes and the human control gene. HPV-negative human sample, a non-template control, and an HPV-positive control (DNA from Ca Ski or HeLa cells) were included in each run. The limit of detection of this assay was 50 HPV DNA copies/mL of blood.

### 2.5. Statistical Analysis

The data were statistically analyzed using GraphPad Prism 9.00 (GraphPad Software, San Diego, CA, USA). The categorical data were analyzed using Fisher’s exact test. The association between *TLR* SNPs and the viral load was estimated using the Mann–Whitney U test. The association of *TLR* SNPs and disease risk was estimated using an odds ratio (OR) with a 95% confidence interval (95% CI). *p*-values of less than 0.05 were statistically significant. The Hardy–Weinberg equilibrium (HWE) and haplotype analyses were performed using the SNPStats Software [56]. Linkage disequilibrium (LD) and haplotype analysis were analyzed by Haploview software version 4.2 (Broad Institute, Cambridge, MA, USA) [57,58]. The Bonferroni correction of the significance level was applied for four multiple comparisons; the significance level for *P^B^* was 0.017 instead of the standard 0.05.

## 3. Results

### 3.1. Frequency of TLR4 and TLR9 Gene Polymorphisms

The *TLR4* 896A > G (rs4986790), 1196C > T (rs4986791), *TLR9* 1486T > C (rs187084), and 1237T > C (rs5743836) SNPs were genotyped in 200 women, including the 70 subjects with OC and 130 healthy women (see Table 1). In the women with OC, the frequencies of genotypes at both the analyzed *TLR4* SNPs and *TLR9* rs187084 were in HWE (*p* = 0.57 for rs4986790, *p* = 0.19 for rs4986790, and *p* = 0.088 for rs187084). In the control group, the frequencies of genotypes at *TLR4* SNPs were in HWE (*p* = 1.000). In contrast, *TLR9* rs5743836 was not in HWE (*p* ≤ 0.050) and was excluded from further analysis.

**Figure 2:**
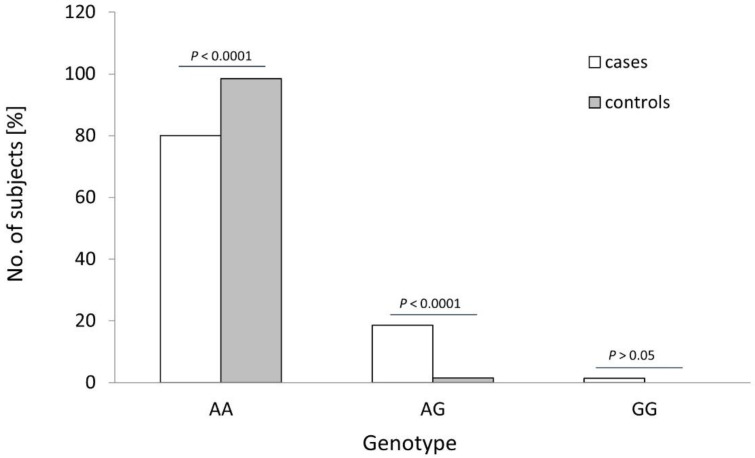
Genotype frequencies of the *TLR4* rs4986790 SNP in patients with ovarian cancer (cases) and healthy women (controls). *p*-values were calculated using Fisher’s exact test.

The distribution of the rs4986790 genotypes of the *TLR4* gene was different between the controls and OC cases (see Table 1 and Figure 2; *p* < 0.0001). The frequency of the wildtype genotype of this SNP was statistically higher in healthy women than in OC cases (98.5% vs. 80.0%; *p* < 0.0001; Fisher’s exact test). The heterozygous variant of this SNP was more frequently found among cancer patients than in healthy individuals (18.6% vs. 1.5%; *p* < 0.0001; Fisher’s exact test). Consequently, the recessive G allele of *TLR4* SNP rs4986790 was detected more frequently in OC patients than in healthy individuals (10.7% vs. 0.8%; *p* < 0.0001; see Table 2). No other differences in the frequency of studied TLRs alleles were observed (*p* > 0.05).

Most individuals (182/200, 91.0%) possessed the wildtype *TLR4* rs4986791 CC genotype, and we did not observe significant differences in the frequency of genotypes in the case and control groups (*p* > 0.05 in all genetical models). Moreover, the heterozygous TC variant of the *TLR9* rs187084 was the most prevalent genotype in both examined groups. No difference was observed in the distribution of both *TLR9* SNPs between the controls and cases (*p* > 0.05).

We determined the frequency of *TLR* SNPs and investigated the association between polymorphisms and the risk of HGSOC incidence. Significant differences in the frequencies of genotypes of *TLR4* rs4986790 were observed between women with HGSOC and controls (Table 3). The AG genotype of this SNP was more frequently observed among HGSOC patients than in controls (25.0% vs. 1.5%; *p* < 0.0001; Fisher’s exact test).

### 3.2. TLR4 Asp299Gly Polymorphism Is Associated with the Increased Risk of Ovarian Cancer

Single-SNP analysis revealed that a heterozygous AG genotype of the *TLR4* SNP rs4986790 was significantly associated with a 14-fold increased risk of OC (OR 14.86, 95% CI 3.24–68.03; *p* < 0.0001 in the codominant model; see Table 1). Moreover, a mutation of a single allele was associated with a 16-fold increased risk of OC (OR 16.00, 95% CI 3.52–72.76; *p* < 0.0001 in the dominant model). This polymorphism was associated with the increased risk of OC even after adjustment for multiple comparisons using Bonferroni’s correction (*P^B^* < 0.0001). Moreover, the women with the heterozygous genotype of rs4986790 also had an approximately 14-fold increased risk of OC disease in an adjusted model that included the HPV16 DNA copy number in the peripheral blood (OR 14.40, 95% CI 2.99–69.41, *p* = 0.0005; Table 1). Similarly, an at least eightfold increased risk of OC was observed in these patients in an adjusted model that included the HPV18 DNAemia level (OR 11.03, 95% CI 2.04–59.72, *p* = 0.003 in the dominant model; Table 1). These associations reached statistical significance after Bonferroni’s correction (*P^B^* < 0.017). No significant association between the *TLR4* rs4986791 or *TLR9* rs187084 genotypes and the risk of OC was found.

The mutation present in at least one allele of the rs4986790 SNP was associated with a twentyfold increased risk of HGSOC occurrence in almost all genetic models (*p* < 0.0001 in the codominant, dominant, and overdominant models; Table 3). The presence of the *TLR4* Asp299Gly polymorphism was also significantly associated with HGSOC in an adjusted model that included the HPV16 DNA copy number (OR 23.27, 95% CI 4.63–116.92; *p* < 0.0001; Table 3). This association reached statistical significance after Bonferroni’s correction for multiple testing.

### 3.3. Associations between TLR SNPs and HPV Infection

HPV16 DNA was detected in the peripheral blood samples collected from 21 of the 70 women with OC (30.0%), whereas HPV18 DNA was found and quantified in 36/70 (51.4%) cancer patients. The viremia levels of HPV16 ranged from 0 to 2.60 × 10^3^ copies/mL (mean 1.32 ± 3.47 × 10^2^ copies/mL), while HPV18 ranged from 0 to 3.40 × 10^3^ copies/mL (mean 2.00 ± 4.57 × 10^2^ copies/mL). Among OC patients, the HPV16 DNA levels in the blood samples were lower in carriers of a wildtype genotype for the *TLR4* rs4986791 compared with those who were heterozygous or homozygous recessive for this polymorphism (*p* = 0.0548; Mann–Whitney U test). No association was observed between the HPV16 and HPV18 DNAemia and any other TLR polymorphisms (*p* > 0.05).

### 3.4. Haplotype Analysis

Multiple-SNP analysis showed that the most common haplotype for the *TLR4* rs4986790 and rs4986791 SNPs, as well as the *TLR9* rs187084, was ACT, which was detected in 59.0% of controls and 44.2% of the OC cases. The GCT haplotype was associated with an increased risk of OC in an unadjusted model (*p* < 0.0001). This haplotype showed an enhanced risk of OC occurrence even after Bonferroni correction for multiple testing (*P^B^* < 0.017).

**Figure 3:**
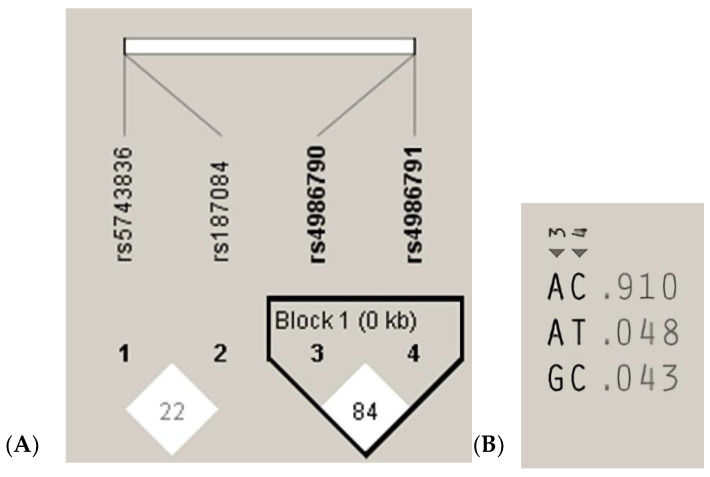
Linkage disequilibrium (LD) block structure of the *TLR9* (1, 2) and *TLR4* (3, 4) SNPs and haplotypes of the *TLR4* gene from the 200 subjects. (**A**), LD plot; (**B**), haplotype analysis.

The measurements of LD (D′) between the analyzed *TLR4* rs4986790, rs4986791 and *TLR9* rs187084, rs5743836 gene variants and 200 cases analyzed in the study are graphically displayed in Figure 3A. The white color indicates LOD < 2, D’ < 1, according to the Standard (D’/LOD) LD color scheme. The numbers in the squares are D′ values multiplied by 100 (|D′| × 100). In the *TLR4* gene, one haplotype block was defined using the Haploview program with default settings, which indicated high LD among the analyzed SNPs (the D’ values between rs4986790 and rs4986791 SNPs are equal to 0.8474) (Figure 3A). Linkage disequilibrium analysis demonstrated that the studied *TLR9* SNPs were not in LD with each other (correlation coefficient r^2^ < 0.2). The analysis of the frequency of haplotypes of the studied *TLR4* gene polymorphisms for all 200 cases (Figure 3B) shows that the AC haplotype for the *TLR4* gene polymorphisms was the common haplotype among the analyzed samples (0.910).

### 3.5. TLR4 Asp299Gly Polymorphism Influences the TLR4 mRNA Expression Level

Then, we investigated the effect of the rs4986790 polymorphism on the expression of the *TLR4* gene. Analysis of *TLR4* gene expression in a subset of the examined patients revealed that the carriers of the heterozygous genotype and minor allele of the *TLR4* SNP rs4986790 did not exhibit significantly different mRNA levels than wildtype SNP genotypes carriers.

## 4. Discussion

To our knowledge, this preliminary study provides the first evidence that Asp299Gly polymorphism in the host *TLR4* gene seems to influence the development of ovarian cancer. The results showed that the heterozygous genotype of the *TLR4* rs4986790 SNP was more common in patients with OC than in healthy women indicating an association of the recessive G allele with an increased risk of OC to its carriers. The patients with the *TLR4* 896A > G polymorphism had an exceptionally higher risk of HGSOC when compared with subjects with the wildtype genotype. So far, no studies have confirmed the association between the *TLR* SNPs and predisposition to ovarian tumorigenesis.

The *TLR4* gene is located in chromosome 9 (9q33.1) and encodes a TLR4 protein of 839 amino acid residues of an approximately 95.7 kDa molecular weight [59,60]. It is known that two non-synonymous polymorphisms located within the third exon of *TLR4*, 896A > G, rs4986790, and 1196C > T, rs4986791, cause the substitution of amino acids Asp299Gly and Thr399Ile, respectively. Both polymorphisms modify the extracellular domain of the TLR4 receptor. It is possible that the replacement of the conserved Asp with Gly at position 299 can disrupt the alpha helix structure of the protein, leading to an extended, less functional beta strain. The results showed that the structural changes influence the binding of ligands in the region of Asp299Gly but not Thr399Ile [61]. A single amino acid substitution of Asp299Gly disturbs the antigenic structure of the extracellular region of the receptor, which may lead to decreased ligand recognition and binding [62]. This can also affect the TLR4 expression and induce an inflammatory response leading to severe tissue destruction [63]. However, TLR4 expression in the normal and neoplastic ovarian epithelium, as well as in FT, has been found [25,64]. Moreover, Asp299Gly polymorphism may affect folding efficiency and protein stability, while the Thr399Ile polymorphism has little effect [61]. *TLR4* polymorphism is also associated with the activation of the cellular signaling pathways that induce the inflammatory response. TLR4 signaling pathways involve the myeloid differentiation primary response gene 88 (MyD88) and the Toll/interleukin 1 receptor (TIR)-domain-containing adapter-inducing interferon-β (TRIF). The MyD88 signaling adaptor gives rise to early activation of NF-κB and pro-inflammatory cytokine release, whereas the TRIF leads to late activation of NF-κB, activation of IRF3, and production of type I interferons and other cytokines [65,66]. *TLR4* Asp299Gly polymorphism impairs TLR4 signaling, as assessed by cytokine production and NF-κB stimulation in response to LPS [67,68,69,70]. In OC patients, the co-expression of TLR4 with MyD88 was associated with a poor prognosis [28,29,71]. In HGSOCs, strong MyD88 expression was associated with the advanced stage of disease and shortened overall survival, while TLR4 expression was not associated with survival [30]. MyD88 is also one of the markers of cancer cell stemness and is among the factors responsible for OC chemoresistance. MyD88-positive OC cells are equated to OC stem cells due to their resistance to pro-apoptotic signals and their ability to create a pro-inflammatory tumor microenvironment [72]. Moreover, MyD88 expression was found to be an unfavorable prognostic factor for OC patients [73,74].

In patients with OC, we identified two non-synonymous *TLR4* SNPs, Asp299Gly and Thr399Ile, at frequencies up to 10.7% and 5.7%, respectively. Moreover, the *TLR4* Asp299Gly polymorphism was detected in 15.0% (6/40) of women with HGSOC. The distribution of Asp299Gly and Thr399Ile was earlier analyzed in 105 Chinese women with OC, but these polymorphisms were not detected in the studied populations [75]. Several studies have confirmed the absence of rs4986790 in the Chinese population and a low prevalence of this SNP in Asian populations [39]. Among Russian patients with OC, Asp299Gly and Thr399Ile polymorphisms were not significantly associated with susceptibility and progression [76]. However, due to the low frequency of the minor allele, it was difficult to detect the effects. Meta-analysis using 22 case-control studies indicated that these *TLR4* polymorphisms were associated with increased cancer risk [39]. Intestinal epithelial Caco-2 cells expressing TLR4-Asp299Gly underwent EMT and morphologic changes associated with tumor progression, whereas cells that expressed wildtype TLR4 did not [77]. Recently, the minor 299Gly (G) and 399Ile (T) alleles were also associated with a significant risk of severe COVID-19 (*p* < 0.001) and higher serum levels of interleukin 6 (IL-6) [78]. Increased levels of IL-6 in OC ascites were found to be an independent predictor of poor survival [79]. It was also found that IL-6 enhances the chemoresistance of OC cells in vitro. IL-6 upregulates the expression of hypoxia-inducible factor (HIF) 1α via the signal transducer and activator of transcription 3 (STAT3) signaling under hypoxia [80].

The TLR9 protein is encoded by the *TLR9* gene located on chromosome 3 (3p21.2). This protein contains 1032 amino acid residues of 115.8 kDa molecular weight [81]. This receptor senses double-stranded DNA molecules containing unmethylated CpG motifs that are common in bacterial or viral genomes. It has been demonstrated that TLR9 was expressed in cancerous ovarian tissues, and this expression was associated with poor differentiation [31]. The correlation of TLR9 expression with the pathological grades may suggest an important role of this receptor in the development and progression of OC [82]. Moreover, TLR9 was expressed both on the membrane and in the cytoplasm of epithelial cells from the ovarian cancer cell line SKOV3 [83]. TLR9 recognizes HPV16 CpG-rich DNA, although its transcription is hindered by the viral E6 and E7 oncoproteins [84,85]. The expression of high-risk HPV oncoproteins deregulates the activity of the NF-κB pathways and decreases *TLR9* expression on the mRNA level [84]. HPV16 E6 and E7 decrease the transcriptional activity of TLR9 and may lead to decreased immune response and escape for HPV16. It was found previously that *TLR9* rs187084, as well as *TLR4* rs4986790 and rs1927911 SNPs, showed an association with HPV16/18 infection in cervical cancer cases [86]. The presence of HPV16 infection with *TLR9* rs352140 SNP increased the risk of cervical cancer [45]. *TLR9* rs5743836 SNP on the promoter region of the *TLR9* gene creates a putative NF-κB-binding site [87]. HPV16 E6 and E7 oncoproteins inhibit NF-κB activity, whereas inhibition of NF-κB promotes cell growth and immortalization [88]. Hence, we tried to explore the correlation between *TLR* polymorphisms and HPV infection. However, we did not find an association between *TLR* SNPs genotypes and the risk of HPV viraemia.

The study had several strengths and limitations. This was the first study to focus on the distribution of the *TLR4* and *TLR9* polymorphisms in women with OC. The strength of the present study was also the clinical evaluation of cancer patients and its important implications. Although the number of OC cases was small for genetic studies, we believe that the Asp299Gly polymorphism was associated with disease progression. Significant differences in genotype distribution in the case-control association study indicated that this polymorphism remained an important risk factor for OC development. Further studies with larger sample sizes are needed to confirm this association. Subsequent studies are required to elucidate the influence of the *TLR4* Asp299Gly polymorphism on cytokine production in OC patients.

## 5. Conclusions

Taken together, our results indicate that the Asp299Gly polymorphism in the *TLR4* gene is associated with the development of ovarian cancer in women, especially in those with the HGSOC subtype. It is possible that this polymorphism conferred reduced secretion of cytokines with antiviral activity and might be a genetic risk factor for the development of OC. The present study demonstrated no association between this polymorphism and HPV16/18 viraemia. An understanding of how the host TLRs mediate cellular signaling and stimulate immune responses is crucial for improving prophylaxis and therapeutic approaches.

## Figures and Tables

**Table 1:** The distribution of genotype frequencies of *TLR* SNPs in healthy women and those with ovarian cancer. An association of *TLR* genotype with the occurrence of ovarian cancer related to HPV infection.

Gene SNP	Model	Genotype	Genotype Frequencies, *n* (%) ^a^		Unadjusted		Adjusted ^b^		Adjusted ^c^	
			Controls	Cases	OR (95% CI)	*p*	OR (95% CI)	*p*	OR (95% CI)	*p*
*TLR4*	Codominant	AA	128 (98.5)	56 (80.0)	1.00	<0.0001	1.00	0.0005	1.00	0.0085
rs4986790		AG	2 (1.5)	13 (18.6)	14.86 (3.24–68.03)		14.40 (2.99–69.41)		8.83 (1.54–50.53)	
		GG	0 (0)	1 (1.4)	NA (0.00–NA)		11.77 (0.00–NA)		NA (0.00–NA)	
	Dominant	AA	128 (98.5)	56 (80)	1.00	<0.0001	1.00	0.0001	1.00	0.003
		AG-GG	2 (1.5)	14 (20)	16.00 (3.52–72.76)		14.40 (2.99–69.41)		11.03 (2.04–59.72)	
	Recessive	AA-AG	130 (100)	69 (98.6)	1.00	0.15	1.00	1	1.00	0.075
		GG	0 (0)	1 (1.4)	NA (0.00–NA)		11.30 (0.00–NA)		NA (0.00–NA)	
	Overdominant	AA-GG	128 (98.5)	57 (81.4)	1.00	<0.0001	1.00	0.0001	1.00	0.013
		AG	2 (1.5)	13 (18.6)	14.60 (3.19–66.81)		14.40 (2.99–69.41)		8.53 (1.49–48.78)	
*TLR4*	Codominant	CC	119 (91.54)	63 (90.0)	1.00	0.35	1.00	0.87	1.00	1
rs4986791		CT	11 (8.5)	6 (8.6)	1.03 (0.36–2.92)		0.71 (0.19–2.64)		1.05 (0.28–3.98)	
		TT	0 (0)	1 (1.4)	NA (0.00–NA)		0.00 (0.00–NA)		0.00 (0.00–NA)	
	Dominant	CC	119 (91.5)	63 (90.0)	1.00	0.72	1.00	0.59	1.00	0.95
		CT-TT	11 (8.5)	7 (10.0)	1. 20 (0.44–3.25)		0.71 (0.19–2.64)		1.05 (0.28–3.98)	
	Recessive	CC-CT	130 (100)	69 (98.6)	1.00	0.15	1.00	1	1.00	1
		TT	0 (0)	1 (1.4)	NA (0.00–NA)		0.00 (0.00–NA)		0.00 (0.00–NA)	
	Overdominant	CC-TT	119 (91.5)	64 (91.4)	1.00	0.98	1.00	0.59	1.00	0.95
		CT	11 (8.5)	6 (8.6)	1.01 (0.36–2.87)		0.71 (0.19–2.64)		1.05 (0.28–3.98)	
*TLR9*	Codominant	TT	35 (28.2)	15 (22.4)	1.00	0.091	1.00	0.14	1.00	0.85
rs187084		TC	81 (65.3)	41 (61.2)	1.18 (0.58–2.41)		0.90 (0.41–1.94)		0.96 (0.40–2.32)	
		CC	8 (6.5)	11 (16.4)	3.21 (1.08–9.57)		2.69 (0.84–8.66)		1.46 (0.32–6.64)	
	Dominant	TT	35 (28.2)	15 (22.4)	1.00	0.38	1.00	0.88	1.00	0.99
		TC-CC	89 (71.8)	52 (77.6)	1.36 (0.68–2.73)		1.06 (0.50–2.23)		1.00 (0.42–2.38)	
	Recessive	TT-TC	116 (93.5)	56 (83.6)	1.00	0.032	1.00	0.049	1.00	0.58
		CC	8 (6.5)	11 (16.4)	2.85 (1.09–7.48)		2.90 (1.02–8.24)		1.50 (0.37–6.01)	
	Overdominant	TT-CC	43 (34.7)	26 (38.8)	1.00	0.57	1.00	0.27	1.00	0.77
		TC	81 (65.3)	41 (61.2)	0.84 (0.45–1.55)		0.68 (0.35–1.35)		0.88 (0.40–1.98)	

^a^ Values are the number of examined healthy women (controls) and those with OC (cases). (%); ^b^ Adjusted analysis was carried out for HPV16 DNA copy number in whole-blood samples; ^c^ Adjusted analysis was carried out for HPV18 DNA copy number in whole-blood samples; OR: odds ratio; 95% CI: 95% confidence interval; *p*, logistic regression model; NA: not available; *P^B^*, The significance level after Bonferroni’s correction for multiple testing was 0.017 (raw *p*-value/3).

**Table 2:** The distribution of the allele frequencies of TLR SNPs in healthy women and women with ovarian cancer.

Gene	SNP	Allele	Allele Frequencies; *n* (%) ^a^		*p*
			Controls	Cases	
*TLR4*	rs4986790	A	258 (99.2)	125 (89.3)	<0.0001
		G	2 (0.8)	15 (10.7)	
	rs4986791	C	249 (95.8)	132 (94.3)	0.623
		T	11 (4.2)	8 (5.7)	
*TLR9*	rs187084	T	151 (60.9)	71 (53.0)	0.158
		C	97 (39.1)	63 (47.0)	
	rs5743836	T	224 (88.9)	125 (90.6)	0.730
		C	28 (11.1)	13 (9.4)	

^a^ Values are the number of alleles (%); *p*-values were calculated using Fisher’s exact test.

**Table 3:** The distribution of genotype frequencies of *TLR* SNPs in healthy women and those with HGSOC subtype.

Gene SNP	Model	Genotype	Genotype Frequencies, *n* (%) ^a^		Unadjusted		Adjusted ^b^		Adjusted ^c^	
			Controls	Cases	OR (95% CI)	*p*	OR (95% CI)	*p*	OR (95% CI)	*p*
*TLR4*	Codominant	AA	128 (98.5)	29 (72.5)	1.00	<0.0001	1.00	0.0001	1.00	0.002
rs4986790		AG	2 (1.5)	10 (25.0)	22.07 (4.59–106.16)		23.27 (4.63–116.92)		13.47 (2.31–78.67)	
		GG	0 (0)	1 (2.5)	NA (0.00–NA)		3.34 (0.00–NA)		NA (0.00–NA)	
	Dominant	AA	128 (98.5)	29 (72.5)	1.00	<0.0001	1.00	<0.0001	1.00	0.0006
		AG-GG	2 (1.5)	11 (27.5)	24.28 (5.10–115.48)		23.27 (4.63–116.92)		16.84 (3.05–93.04)	
	Recessive	AA-AG	130 (100)	39 (97.5)	1.00	0.088	1.00	1	1.00	0.053
		GG	0 (0)	1 (2.5)	NA (0.00–NA)		2.42 (0.00–NA)		NA (0.00–NA)	
	Overdominant	AA-GG	128 (98.5)	30 (75.0)	1.00	<0.0001	1.00	<0.0001	1.00	0.0037
		AG	2 (1.5)	10 (25.0)	21.33 (4.44–102.48)		23.27 (4.63–116.92)		12.80 (2.20–74.52)	
*TLR4*	Codominant	CC	119 (91.54)	37 (92.5)	1.00	0.18	1.00	0.58	1.00	0.74
rs4986791		CT	11 (8.5)	2 (5.0)	0.58 (0.12–2.76)		0.37 (0.05–3.01)		0.47 (0.06–3.82)	
		TT	0 (0)	1 (2.5)	NA (0.00–NA)		0.00 (0.00–NA)		0.00 (0.00–NA)	
	Dominant	CC	119 (91.5)	37 (92.5)	1.00	0.85	1.00	0.29	1.00	0.44
		CT-TT	11 (8.5)	3 (7.5)	0.88 (0.23–3.31)		0.37 (0.05–3.01)		0.47 (0.06–3.82)	
	Recessive	CC-CT	130 (100)	39 (97.5)	1.00	0.088	1.00	1	1.00	1
		TT	0 (0)	1 (2.5)	NA (0.00–NA)		0.00 (0.00–NA)		0.00 (0.00–NA)	
	Overdominant	CC-TT	119 (91.5)	38 (95.0)	1.00	0.45	1.00	0.29	1.00	0.44
		CT	11 (8.5)	2 (5.0)	0.57 (0.12–2.68)		0.37 (0.05–3.01)		0.47 (0.06–3.82)	
*TLR9*	Codominant	TT	35 (28.2)	12 (30.8)	1.00	0.094	1.00	0.13	1.00	0.72
rs187084		TC	81 (65.3)	20 (51.3)	0.72 (0.32–1.63)		0.60 (0.25–1.49)		0.70 (0.27–1.84)	
		CC	8 (6.5)	7 (17.9)	2.55 (0.76–8.54)		2.19 (0.58–8.19)		1.09 (0.19–6.17)	
	Dominant	TT	35 (28.2)	12 (30.8)	1.00	0.76	1.00	0.51	1.00	0.53
		TC-CC	89 (71.8)	27 (69.2)	0.88 (0.40–1.94)		0.75 (0.32–1.77)		0.74 (0.29–1.89)	
	Recessive	TT-TC	116 (93.5)	32 (82.0)	1.00	0.043	1.00	0.084	1.00	0.70
		CC	8 (6.5)	7 (17.9)	3.17 (1.07–9.41)		3.02 (0.91–10.04)		1.38 (0.27–6.96)	
	Overdominant	TT-CC	43 (34.7)	19 (48.7)	1.00	0.12	1.00	0.092	1.00	0.42
		TC	81 (65.3)	20 (51.3)	0.56 (0.27–1.16)		0.50 (0.22–1.12)		0.69 (0.28–1.70)	

^a^ Values are the number of examined healthy women (controls) and those with HGSOC (cases). (%); ^b^ Adjusted analysis was carried out for HPV16 DNA copy number in whole-blood samples; ^c^ Adjusted analysis was carried out for HPV18 DNA copy number in whole-blood samples; OR: odds ratio; 95% CI: 95% confidence interval; *p*, logistic regression model; NA: not available; *P*^B^. The significance level after Bonferroni’s correction for multiple testing was 0.017 (raw *p*-value/3).

## Data Availability

The data generated during the current study are available from the corresponding author upon reasonable request.
